# The incidence of relative adrenal insufficiency in patients with septic shock after the administration of etomidate

**DOI:** 10.1186/cc4979

**Published:** 2006-07-19

**Authors:** Zulfiqar Mohammad, Bekele Afessa, Javier D Finkielman

**Affiliations:** 1 Department of Cardiothoracic Anesthesiology, The Cleveland Clinic, 9500 Euclid Ave., Cleveland, OH 44195, USA; 2 Division of Pulmonary and Critical Care Medicine, Mayo Clinic College of Medicine, 200 First Street SW, Rochester, MN 55905, USA; 3 Intensive Care Unit, Saint Alexius Medical Center, 900 East Broadway Ave., Bismarck, ND 58501, USA

## Abstract

**Introduction:**

Etomidate blocks adrenocortical synthesis when it is administered intravenously as a continuous infusion or a single bolus. The influence of etomidate administration on the incidence of relative adrenal insufficiency in patients with septic shock has not been formally investigated. The objective of this study was to determine the incidence of relative adrenal insufficiency in patients with septic shock after etomidate administration compared with patients with septic shock who did not receive etomidate.

**Methods:**

In this retrospective study, 152 adults with septic shock who had a consyntropin stimulation test between March 2002 and August 2003 in a tertiary medical center were included. Relative adrenal insufficiency was defined as a rise in serum cortisol ≤ 9 μg/dl after the administration of 250 μg of consyntropin. Patients were divided into those who did and those who did not receive etomidate before the stimulation test. The proportion of patients with relative adrenal insufficiency in these two groups was compared using Fischer's exact test. A *P *of value < 0.05 was considered statistically significant.

**Results:**

The mean age of the patients was 64 years, 59% of patients were male, 97% of patients were white and their hospital mortality rate was 57%. Thirty-eight patients (25%) received etomidate before the cosyntropin stimulation test, and the median (interquartile range) time interval between the administration of the drug and the test was 7 (4–10) hours. The incidence of relative adrenal insufficiency was 76% in the patients who received etomidate compared with 51% in the patients who did not (*P *= 0.0077).

**Conclusion:**

The incidence of relative adrenal insufficiency in patients with septic shock is increased when the stimulation test is performed after the administration of etomidate.

## Introduction

Relative adrenal insufficiency, defined as a blunted increase in cortisol levels after stimulation with adrenocorticotrophic hormone, is common in patients with septic shock [1], and replacement therapy with hydrocortisone and fludrocortisone has been shown to reduce the risk of death in this setting [2]. Etomidate, an induction agent widely used to facilitate endotracheal intubation, is an imidazole derivative that reversibly blocks adrenocortical synthesis when administered intravenously as a continuous infusion or a single bolus [3]. A single dose of etomidate is a major risk factor for the development of relative adrenal insufficiency for at least 24 hours after its administration [4]. Because most patients with septic shock require endotracheal intubation, and the influence of etomidate administration on the incidence of relative adrenal insufficiency in patients with septic shock has not been formally investigated, the objective of this study was to determine the incidence of relative adrenal insufficiency in patients with septic shock after etomidate administration. We hypothesised that the administration of etomidate increases the incidence of relative adrenal insufficiency in patients with septic shock.

## Materials and methods

In this retrospective study, we first identified 1,207 consecutive patients who had their serum cortisol level measured at the Mayo Clinic (Rochester, MN, USA) between March 2002 and August 2003. The records of these patients were reviewed. Only adults with septic shock who had a short cosyntropin stimulation test were included in this study. In this test, cortisol levels were measured immediately before and both 30 minutes and 60 minutes after the administration of cosyntropin (250 μg). The cortisol response was calculated as the difference between the highest and the lowest cortisol levels measured before and after the administration of cosyntropin. Relative adrenal insufficiency was defined as a rise in serum cortisol ≤ 9 μg/dl [1]. Septic shock was defined according to the American College of Chest Physicians/Society of Critical Care Medicine Consensus Conference Committee criteria [5]. Patients were excluded if they had received corticosteroids or ketoconazole before the cosyntropin stimulation test, or if they had an adrenal or pituitary disorder. The Mayo Foundation Institutional Review Board approved the study, and a waiver of informed consent was granted. Patients who did not authorize their medical records to be reviewed for research were excluded. Data collected included demographics, the results of the cosyntropin stimulation test, the use of etomidate, the time interval between the etomidate administration and the cosyntropin stimulation test, the presence of relative adrenal insufficiency, the use of corticosteroids or ketoconazole before the test and the presence of adrenal or pituitary disorders. Descriptive data were summarized as mean (± standard deviation (SD)), median (with interquartile range (IQR)) or percentages. Patients were divided into those who did and those who did not receive etomidate before the cosyntropin stimulation test. The student's *t* test, the Mann-Whitney *U *test and Fischer's exact test were used for comparisons between groups. The 95% confidence intervals (95% CI) were calculated, as needed. A *P *value < 0.05 was considered statistically significant.

## Results

Among the 1,207 patients who had their cortisol level measured, 163 patients with septic shock had a cosyntropin stimulation test. After excluding 10 patients who had received corticosteroids before the test and one patient who had an adrenal tumor, 152 patients were included in this study.

The mean age (± SD) of the patients was 64 (± 17) years, 59% of patients were male and 97% of patients were white. None of the patients had received ketoconazole or had history of a pituitary disorder. The hospital mortality rate was 57%. Thirty-eight patients (25%) received etomidate before the cosyntropin stimulation test. The median (IQR) time interval between the administration of the drug and the test was 7 (4–10) hours. No significant difference in the baseline characteristics was found in the patients who received etomidate compared with those who did not (Table 1). The incidence of relative adrenal insufficiency was 76% (95% CI, 67–87%) in patients who received etomidate compared with 51% (95% CI, 42–60%) in patients who did not receive etomidate (*P *= 0.0077; Table 1).

## Discussion

In this study, three-quarters of the patients with septic shock who received etomidate had relative adrenal insufficiency compared with only half of the patients with septic shock who did not receive etomidate.

A previous study showed that 54% (95% CI, 47–61%) of patients with septic shock had relative adrenal insufficiency [1], which is similar to our findings. In critically ill patients, a single dose of etomidate suppresses adrenal function for approximately 24 hours [4,6]. Not surprisingly, we found that 76% (95% CI, 67–87%) of our patients with septic shock had relative adrenal insufficiency when tested after the administration of etomidate. Interestingly, in the study by Annane *et al*. [2], in which low doses of hydrocortisone and fluodrocortisone reduced the risk of death in patients with septic shock and relative adrenal insufficiency, 72 patients received etomidate preceding the stimulation test. Of these 72 patients, 68 patients (94%) had relative adrenal insufficiency [2,7].

Our study has some limitations. It was performed in a single tertiary medical center and has a retrospective design. Moreover, the number of patients who received etomidate was relatively small. The smallness of the sample size might account for absence of statistically significant differences in some of the variables, including baseline serum cortisol level and hospital mortality rate, between patients who did and did not receive etomidate. In spite of these limitations, our findings highlight the importance of the ongoing controversies and discussions about the use of etomidate as an induction agent for endotracheal intubation in the intensive care unit, particularly in patients with septic shock, because of the high frequency of relative adrenal insufficiency provoked by this medication [3,8,9]. Two alternative approaches have been proposed to resolve the etomidate controversy. One option is to eliminate the use of etomidate altogether in patients with septic shock, and use alternative agents [3,9]. The second option is to start treatment with corticosteroids after the administration of etomidate [3,8]. Whether there is a real need to test for adrenal insufficiency, on the basis that our study shows that most patients with septic shock had relative adrenal insufficiency after the administration of etomidate, and what dose of corticosteroids should be administered are questions that must be studied in prospective trials.

## Conclusion

Relative adrenal insufficiency is present in most patients with septic shock after the administration of etomidate. Caution is recommended when using etomidate in these patients.

## Key messages

• 	Etomidate reversibly blocks adrenocortical synthesis when administered intravenously as a continuous infusion or a single bolus.

• 	The majority of patients with septic shock show evidence of relative adrenal insufficiency when the cosyntropin stimulation test is performed within 24 hours of the administration of etomidate.

## Abbreviations

CI = confidence interval; IQR = interquartile range; SD = standard deviation.

## Competing interests

The authors declare that they have no competing interests.

## Authors' contributions

ZM was involved in the conception and design of the study, acquisition and interpretation of the data and drafting the manuscript; BA was involved in the conception and design of the study, analysis and interpretation of the data and critical revision of the manuscript; and JDF was involved in the conception and design of the study, analysis and interpretation of the data, statistical analysis and drafting the manuscript. All authors read and approved the final manuscript.

## Figures and Tables

**Table 1:** Differences between patients with septic shock who did and did not receive etomidate

	Etomidate **(*n *= 38)**	**No etomidate (*n *= 114)**	***P***
Mean age (± SD), years	63 (± 18)	65 (± 17)	0.5321
Male gender, *n *(%)	20 (53)	69 (61)	0.4487
White, *n *(%)	37 (97)	111 (97)	1.0000
Hospital mortality rate, *n *(%)	24 (63)	63 (55)	0.4517
Median baseline cortisol level (IQR), μg/dl	32 (23–45)	26 (20–42)	0.3492
Median 30-minute cortisol level (IQR), μg/dl	36 (26–47)	36 (26–53)	0.7642
Median 60-minute cortisol level (IQR), μg/dl	38 (28–52)	41 (28–57)	0.6674
Relative adrenal insufficiency, *n *(%)	29 (76)	58 (51)	0.0077

IQR, interquartile range; SD, standard deviation.
